# Supplementary material for: Cytoplasmic NAD/H synthesis via NRK1 regulates inflammatory capacity and promotes survival of CD4+ T cells
Source: Nat Commun. 2026 Feb 4;17:2349. doi: 10.1038/s41467-026-68863-w (PMC12979809; doi:10.1038/s41467-026-68863-w)
Supplement: Supplementary file 1 — Supplementary Information [file 41467_2026_68863_MOESM1_ESM.pdf]

Cytoplasmic NAD/H synthesis via NRK1 regulates inflammatory capacity and promotes survival of CD4⁺ T cells

[1,2]Victoria Stavrou, [1,2]Myah Ali, [1,2,3]Nancy Gudgeon, [1,2]Emma L Bishop, [1,2]Taylor Fulton-Ward, [1,2]Bethany Turley, [2]Silke Heising, [1,4]Sally H Mohamed, [1,4]Sofia Hain, [1,4]Lorna George, [5]Minghao Deng, [6,7]Jack McCowan, [1,4]Lozan Sheriff, [1]Scott P Davies, [2]Bryan Marzullo, [2]Daniel A Tennant, [5]Craig L Doig, [1]David A Bending, [6,7]Ed W Roberts, [5]Gareth G Lavery, [1,4]Rebecca A Drummond and [1,2*]Sarah Dimeloe

[1] Department of Immunology and Immunotherapy, College of Medicine and Health, University of Birmingham, Birmingham, UK.

[2] Department of Metabolism and Systems Science, College of Medicine and Health, University of Birmingham, Birmingham, UK.

[3] Institute of Cancer and Genomic Sciences, College of Medical and Dental Sciences, University of Birmingham, Birmingham, UK.

[4] Institute of Microbiology and Infection, College of Medical and Dental Sciences, University of Birmingham, Birmingham, UK.

[5] Department of Biosciences, School of Science and Technology, Nottingham Trent University, Nottingham, UK.

[6] Cancer Research UK Scotland Institute, Glasgow, UK.

[7] School of Cancer Sciences, University of Glasgow, Scotland, UK

Supplementary Material

Supplementary Figures 1-10

Table S1

Table S2

Table S3

Supplemental Methods

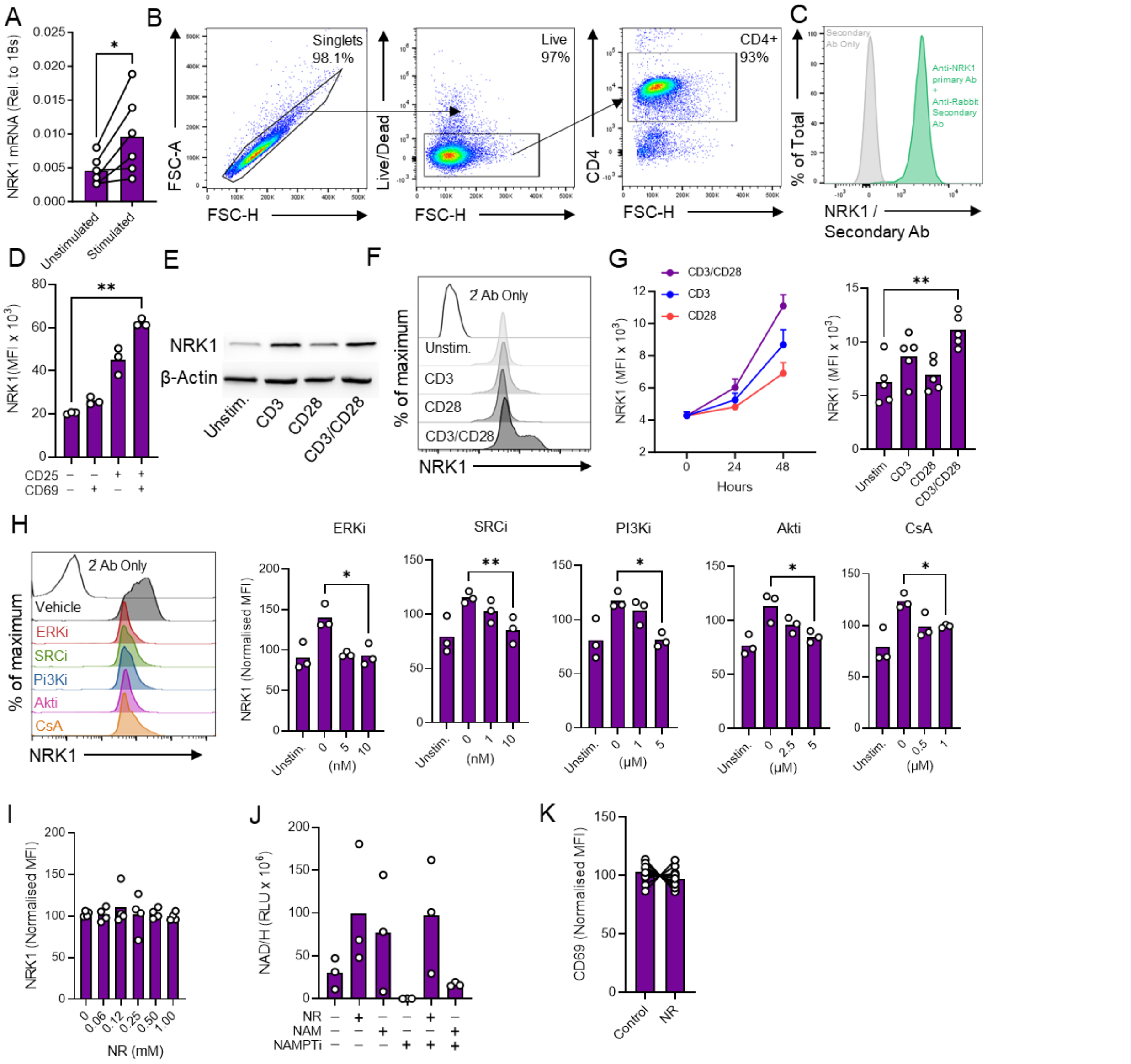

Supplementary Figure 1: (Related to Figure 1)

(**A**) Human CD4^{+} T cells were stimulated via CD3/CD28 for 48 hours and assessed for NRK1mRNA abundance by qPCR (expressed relative to 18S for n=6 independent donors). (**B**) Example gating strategy for flow cytometry analysis of purified human CD4^{+} T cell cultures in Figure 1B, E, G-H. (**C**) Example flow cytometry staining of human CD4^{+} T cells with anti-NRK1 primary and appropriate secondary antibody compared to staining with secondary antibody only. (**D**) Human CD4^{+} T cells were stimulated as in (A) and NRK1 expression assessed by flow cytometry alongside surface staining of the activation markers CD25 and CD69 (summarised NRK1 mean fluorescence intensity (MFI) within indicated populations for n=3 independent donors). (**E-G**) Human CD4^{+} T cells were stimulated with CD3 and/or CD28 as indicated, for time points indicated (G) and assessed for protein abundance by (**E**) western blot and (**F-G**) flow cytometry (E-F, one representative experiment; G, summary data for n=5 independent donors). (**H**) Human CD4^{+} T cells were stimulated via CD3/CD28 for 48 hours in presence of indicated inhibitors (or DMSO vehicle control in “0”) and assessed for NRK1 protein abundance by flow cytometry (representative histograms and summarised for n=3 independent donors). (**I**) Human CD4^{+} T cells were stimulated via CD3/CD28 for 48 hours in presence of NR at indicated concentrations and assessed for NRK1 protein abundance by flow cytometry (summarised for n=4 independent donors). (**J**) Human CD4^{+} T cells were stimulated as in (H) in presence of indicated compounds (0.5mM NR or NAM, 0.1µM NAMPTi) and assayed for total NAD/H abundance (summarised for n=3 independent donors). (**K**) Human CD4^{+} T cells were stimulated as in (H) and assessed for CD69

expression by flow cytometry (summarised for n=12 independent donors). Where normalised, data are expressed as a percentage of the average (mean) value across all samples analysed for each individual donor. p values were calculated by (A) paired t test, (D,G-H,) repeated measures ANOVA with Holm-Sidak post-hoc test and (K) paired t test. * $p < 0.05$, ** $p < 0.01$. (A) p=0.0111, (D) p=0.0030, (G) p=0.0077, (H) p=0.0363, p=0.0096, p=0.0427, p=0.0383, p=0.0202. Source data are provided as a Source Data file.

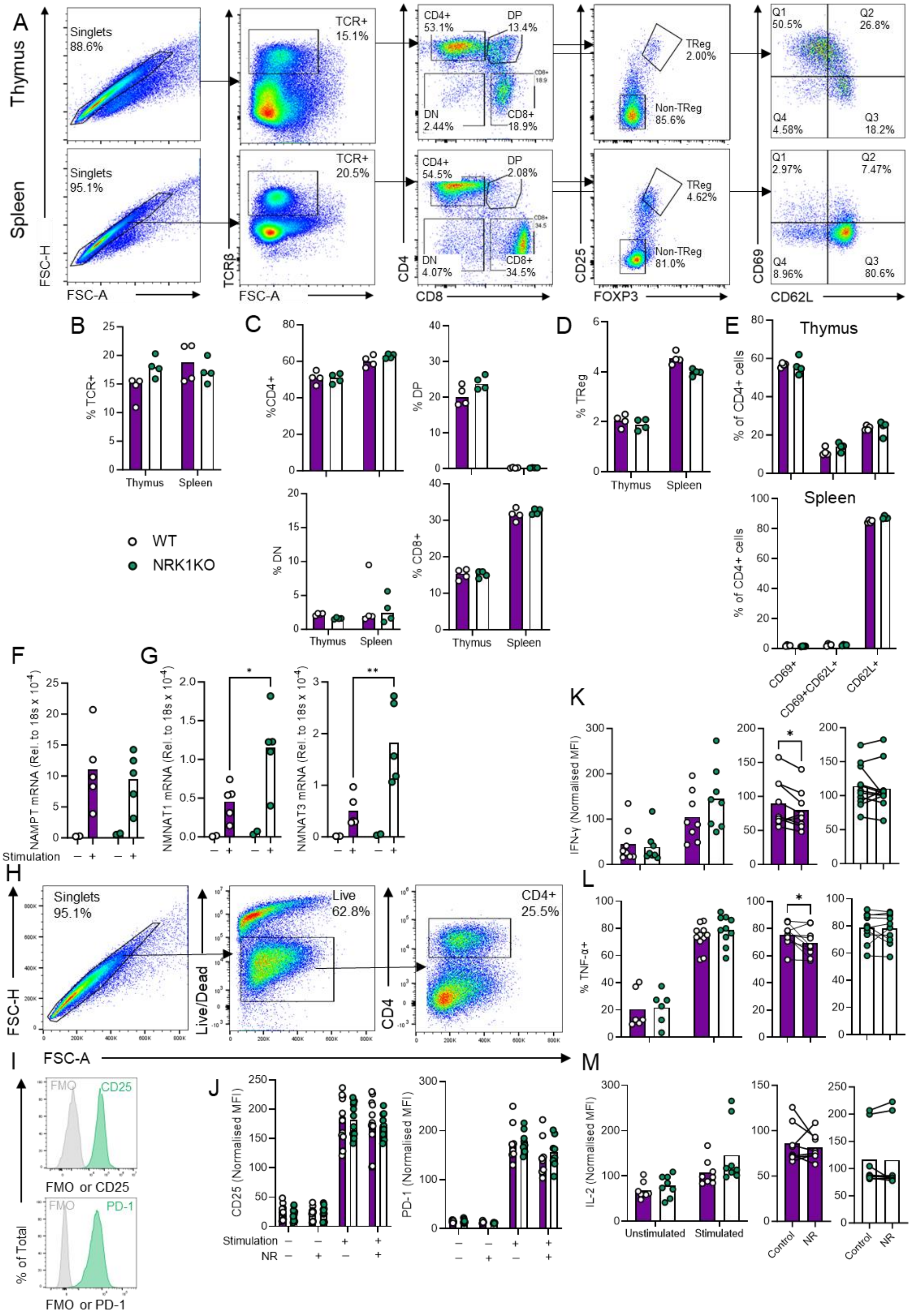

Supplementary Figure 2: (Related to Figure 2)

(**A-E**) Total murine thymocytes or splenocytes of littermate WT or NRK1KO animals as indicated were analysed ((A) example of gating strategy) for (**B**) total T cells, (**C**) CD4$^+$, CD8$^+$, double negative (DN) and double positive (DP) T cell populations, (**D**) regulatory T cells (TReg) and (**E**) maturation status of CD4$^+$ T cells by flow cytometry (representative flow cytometry plots to show gating strategy and summarised data from n=4 individual animals per group). (**F-G**) Murine CD4$^+$ T cells were isolated from spleens of littermate WT or NRK1KO animals, cultured ± stimulation via CD3/CD28 for 48 hours and assessed for (**F**) NAMPT and (**G**) NMNAT1 or NMNAT3 transcript abundance by qPCR (summarised for n=5 individual animals per group, shown relative to 18s). (**H-M**) Murine CD4$^+$ T cells were isolated from spleens of littermate WT or NRK1KO animals as indicated, cultured ± stimulation via CD3/CD28 and ± 0.5mM

NR for 48 hours and assessed (H, example of gating strategy; I, examples of fluorescence minus one (FMO) controls) for abundance of (**J**) surface CD25 and PD-1 expression, (**K**) IFN-γ, (**L**) TNF-α and (**M**) IL-2 by intracellular cytokine staining and flow cytometry (summarised for J: (left) n=12 individual animals per group (right) n=8 individual animals per group; K: n=8 individual animals per group, L n=11 individual animals per group, M n=8 individual animals per group). Where normalised, data are expressed as a percentage of the average (mean) value across all samples analysed together for a batch of mice, of equivalent numbers of WT and NRK1KO. p values were calculated by (A-G, K-M (left panels)) two-way ANOVA with Holm-Sidak post-hoc test and (K-M (right panels) by paired t test. * $p < 0.05$. (G) p=0.0212, p=0.0058, (K) p=0.0491, (L) p=0.0377. Source data are provided as a Source Data file.

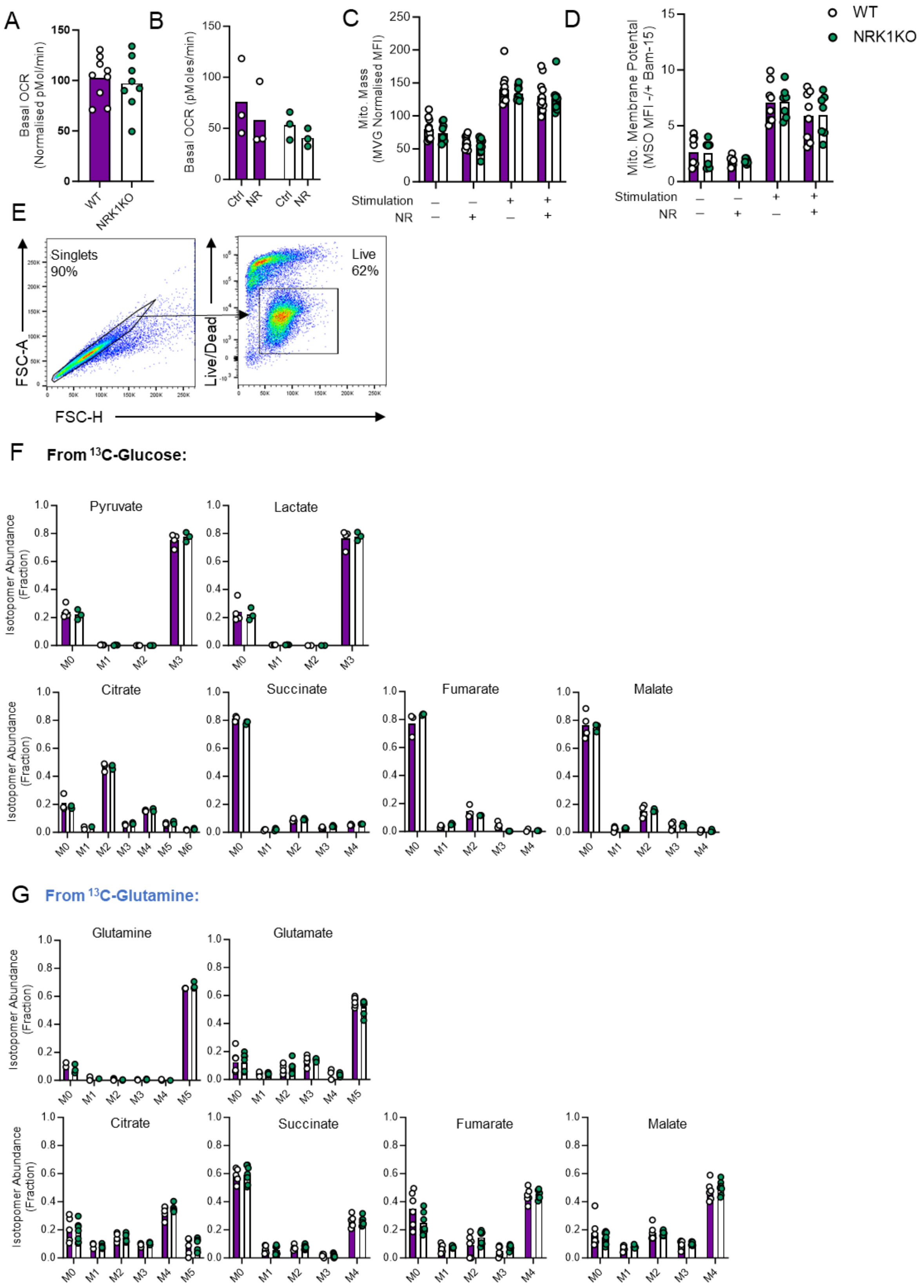

Supplementary Figure 3: (Related to Figure 3)

(**A-B**) Murine CD4$^+$ T cells were isolated from spleens of littermate WT or NRK1KO animals as indicated, stimulated via CD3/CD28 for 48 hours +/- 0.5mM NR as indicated and assessed by extracellular flux analysis for basal oxygen consumption rates (OCR, summarised for (A) n=8 and (B) n=3 individual animals per group) (**C-D**) Murine CD4$^+$ T cells isolated and stimulated as in (A) were assessed for (**C**) mitochondrial mass with mitoview green and (**D**) mitochondrial membrane potential with mitospy orange, expressed as a ratio of MSO MFI in absence of the mitochondrial uncoupler Bam-15 over MSO MFI in presence of Bam-15 (summarised for n=10 WT and n=11 NRK1KO animals) (**E**) Example of gating strategy for mitoview/mitospy and puromycin analysis in Figure 3I. (**F-G**) Murine CD4$^+$ T cells isolated as in (A) were stimulated for 24 hours in presence of fully labelled ^{13}C-glucose or ^{13}C-glutamine and

assessed for mass isotopomer distribution of indicated metabolites by GC-MS (n=4 individual animals per group). Where normalised, data are expressed as a percentage of the average (mean) value across all samples analysed together for a batch of mice, of equivalent numbers of WT and NRK1KO. p values were calculated by (A) unpaired t test and (B-F) two-way ANOVA and Holm-Sidak post-hoc test. Source data are provided as a Source Data file.

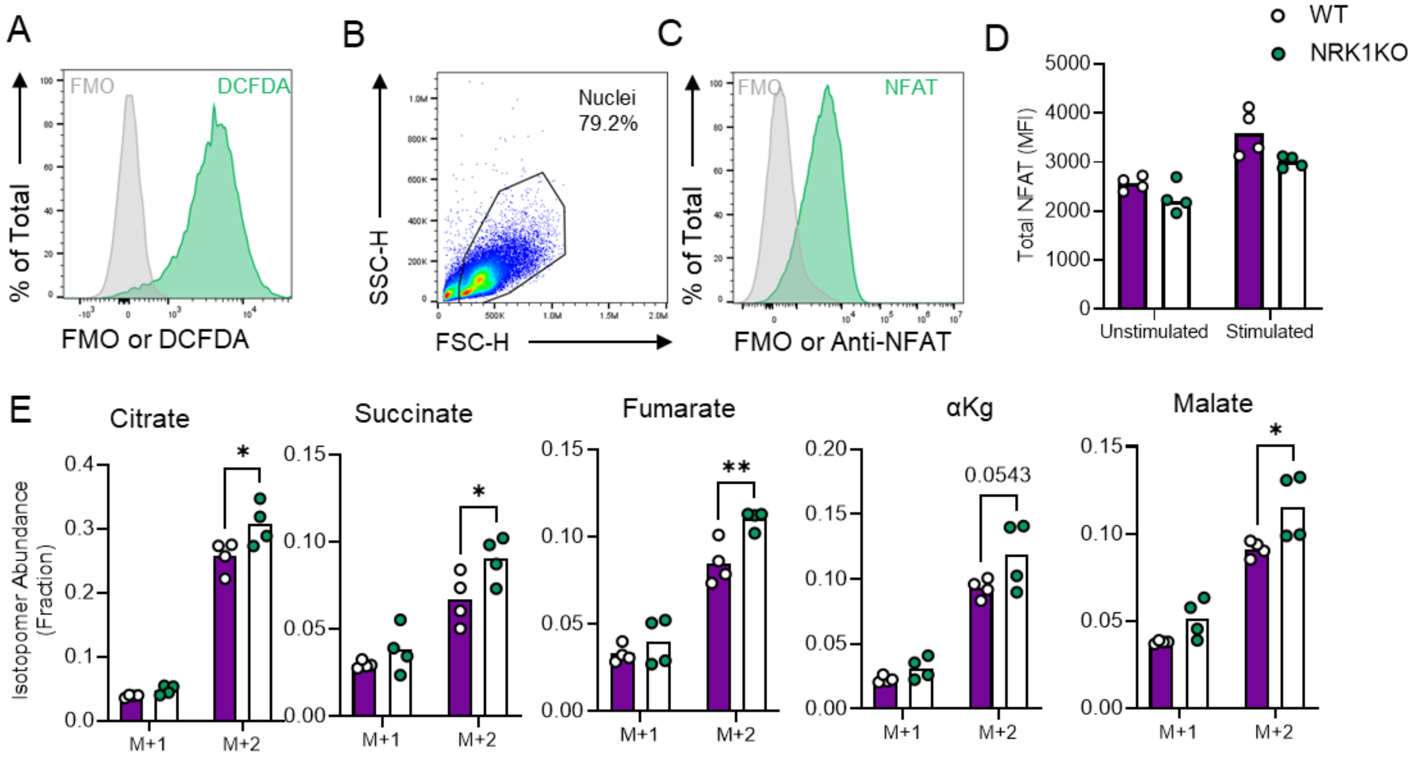

Supplementary Figure 4: (Related to Figure 4)

(**A**) Example of fluorescence minus one (FMO) staining for DCFDA analysis by flow cytometry. (**B**) Example of gating strategy for nuclear NFAT analysis in Figure 4F. (**C**) Example of fluorescence minus one (FMO) staining for DCFDA analysis by flow cytometry. (**D**) Murine CD4^{+} T cells were isolated from spleens of littermate WT or NRK1KO animals as indicated, cultured ± stimulation via CD3/CD28 and ± 0.5mM NR for 48 hours and assessed for abundance of NFAT within whole fixed cells by intracellular flow cytometry (summarised for n=4 individual animals per group). (**E**) Murine CD4^{+} T cells isolated as in (A) were stimulated for 24 hours in presence of 1,2 labelled ^{13}C-glucose and assessed for mass isotopomer distribution of indicated metabolites by GC-MS (summarised for n=4 individual animals per group). p values were calculated by (D-E) two-way ANOVA and Holm-Sidak's post-hoc test. * p < 0.05, ** p < 0.01. (E) p=0.0108, p=0.0356, p=0.0066, p=0.0201. Source data are provided as a Source Data file.

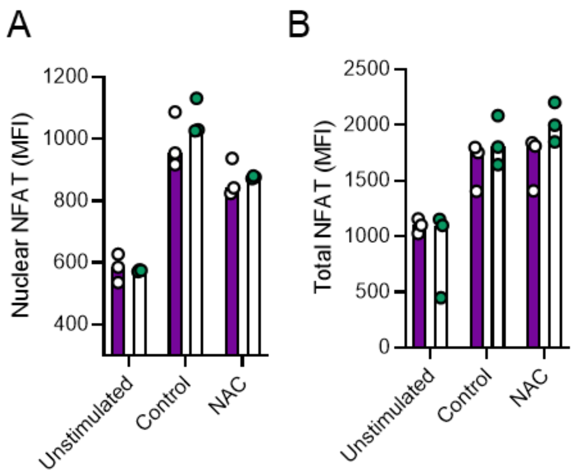

Supplementary Figure 5: (Related to Figure 5)

(**A-B**) Murine CD4$^+$ T cells were isolated from spleens of littermate WT or NRK1KO animals as indicated, cultured ± stimulation via CD3/CD28 and ± N-acetylcysteine (NAC, 5mM) for 48 hours and analysed for (A) nuclear and (B) total cellular NFAT by flow cytometry (summarised for n=3 individual animals per group). Source data are provided as a Source Data file.

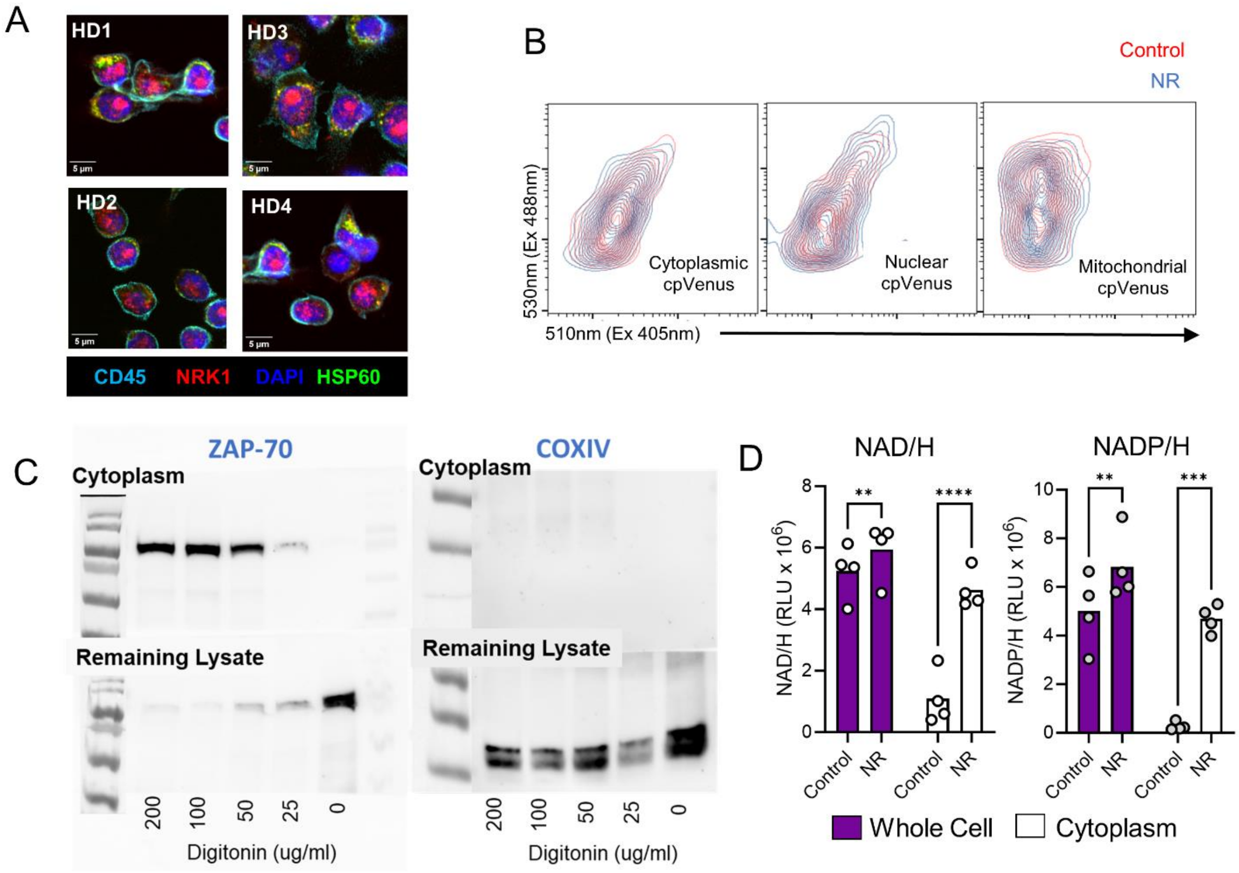

Supplementary Figure 6: (Related to Figure 6)

(**A**) Confocal microscopy was performed on of human CD4$^+$ T cells stimulated via CD3/CD28 for 48 hours stained for NRK1, DAPI (nuclear marker), CD45 (cell membrane) and HSP60 (mitochondrial marker), images of n=4 independent healthy donors (HD). (**B**) Representative flow cytometry plots of cpVenus control sensors transfected into activated human CD4$^+$ T cells treated with indicated compounds for 8 hours. (**C**) Human CD4$^+$ T cells were stimulated via CD3/CD28 for 48 hours, then treated with indicated concentration of digitonin for 4 minutes to permeabilise the plasma membrane and assessed for ZAP-70 (cytoplasmic) or COXIV (mitochondrial) protein abundance within indicated cell fractions by western blot. (**D**) Human CD4$^+$ T cells isolated as in (A) were stimulated via CD3/CD28 for 48 hours ± 0.5mM NR, then left intact or treated with 50µg/ml digitonin for 4 minutes to permeabilise the plasma membrane and assessed for NAD/H and NADP/H abundance within intact, whole cells or cytoplasmic fractions – expressed as raw RLU for each assay (summarised for n=4 individual donors). p values were calculated by (D) two-way ANOVA and Holm-Sidak's post-hoc test. * $p < 0.05$, ** $p < 0.01$. (D) p=0.0051, p<0.0001, p=0.0027, p=0.0004. Source data are provided as a Source Data file.

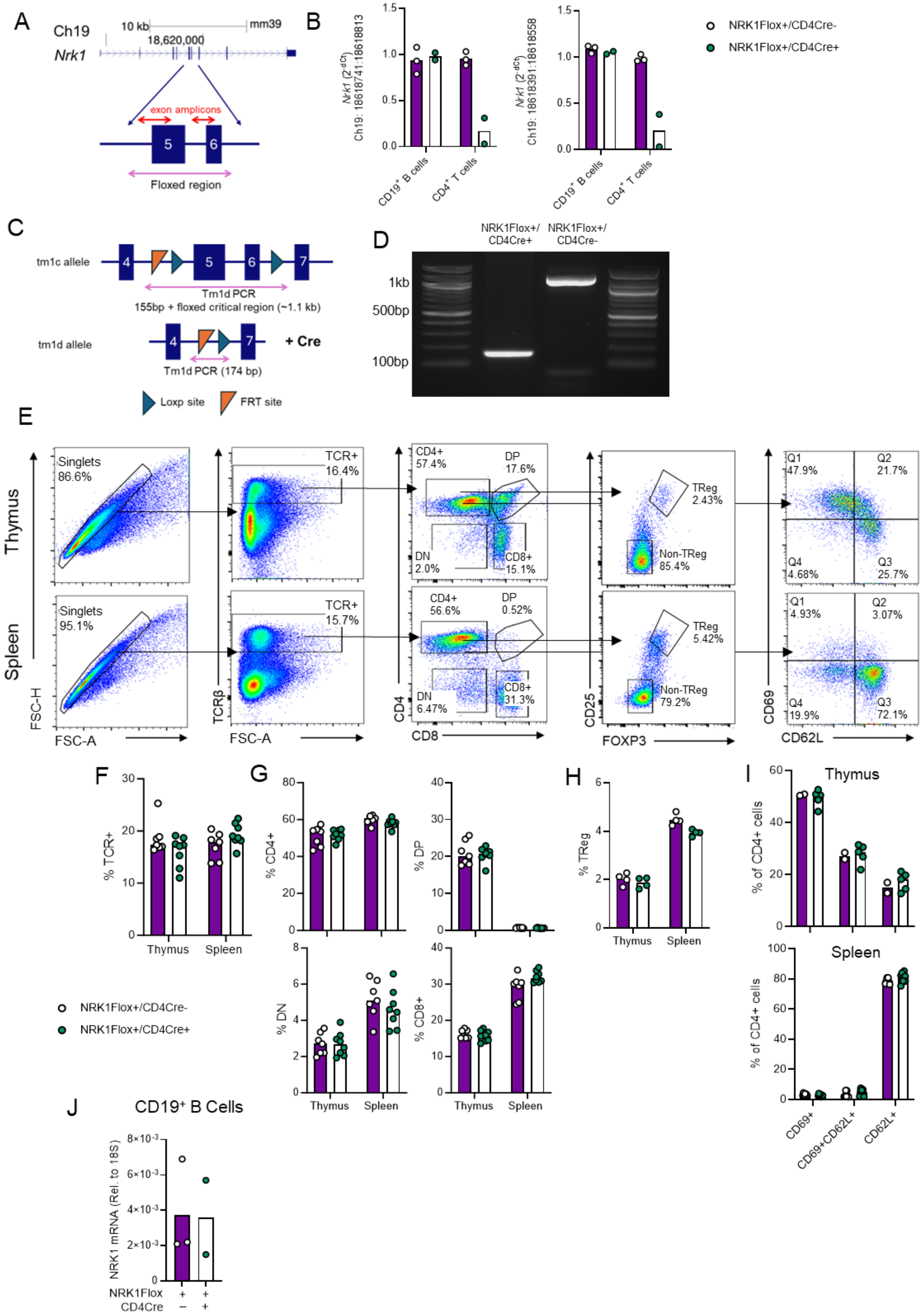

Supplementary Figure 7: (Related to Figure 7) (**A**) Diagram showing the region of the Nrk1 gene targeted for Flox/Cre mediated deletion and mapping of genomic DNA primers employed (**Table S3**). (**B**) Genomic DNA was isolated from the indicated cell populations, FACS-sorted from splenocytes of T cell-specific NRK1KO mice

(NRK1Flox+/CD4Cre+) or littermate Cre-recombinase negative controls (NRK1Flox+/CD4Cre-) animals and assessed by qPCR for abundance of the regions indicated, expressed relative to an untargeted region within the *Il2ra* gene (data summarised for n=3 NRK1Flox+/CD4Cre- and n=2 NRK1Flox+/CD4Cre+ animals). (**C**) Diagram showing the Floxed *Nrk1* region ("tm1c" allele) and Cre-mediated recombined "tm1d" allele, and the subsequent expected PCR products from end-stage PCR using primers covering the Floxed region. (**D**) Genomic DNA isolated from bead-sorted $CD4^+$ T cells from splenocytes of T cell-specific NRK1KO (NRK1Flox+/CD4Cre+) or littermate Cre-recombinase negative control (NRK1Flox+/CD4Cre-) mice was assessed by end-stage PCR for presence of the Cre-mediated recombined or full-length *Nrk1* alleles indicated in (C). (**E-I**) Total murine thymocytes or splenocytes of T cell-specific NRK1KO mice (NRK1Flox+/CD4Cre+) or littermate Cre-recombinase negative controls (NRK1Flox+/CD4Cre-) animals as indicated were analysed (E, example of gating strategy) for (**F**) total T cells, (**G**) $CD4^+$, $CD8^+$, double negative (DN) and double positive (DP) T cell populations, (**H**) regulatory T cells (TReg) and (**I**) maturation status of $CD4^+$ T cells by flow cytometry (representative flow cytometry plots to show gating strategy and summarised data from n=7 individual animals per group). (**J**) mRNA was isolated from $CD19^+$ B cells, FACS-sorted from indicated animals and stimulated for 24 hours with 50ng/ml LPS, and assessed for NRK1 transcript abundance, expressed relative to 18s (data summarised for n=3 NRK1Flox+/CD4Cre- and n=2 NRK1Flox+/CD4Cre+ animals). p values were calculated by two-way ANOVA and Holm-Sidak's post-hoc test. * $p < 0.05$, ** $p < 0.01$. Source data are provided as a Source Data file.

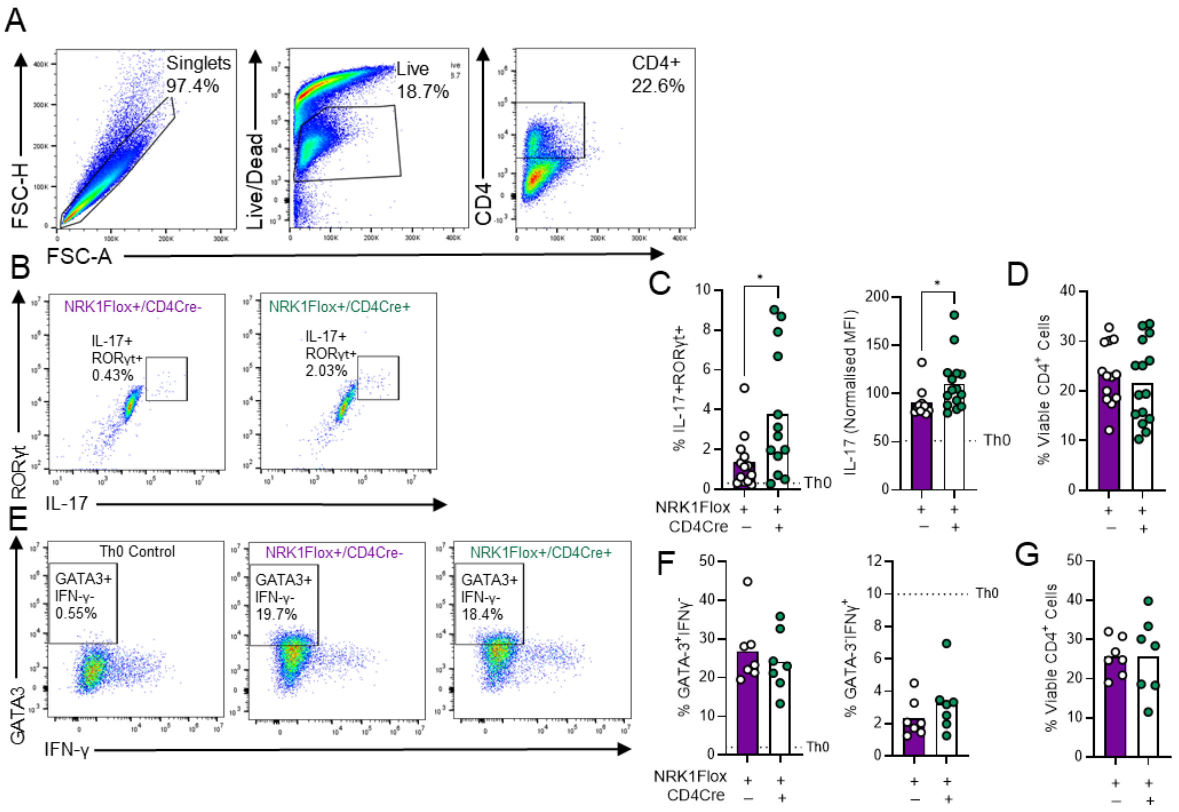

Supplementary Figure 8: (Related to Figure 7)

(**A**) Example of gating strategy for analysis of Th1, Th17 and Th2 polarised cells in Figure 7E-H and Supp Fig 7 B-G. (**B-G**) Murine CD4$^+$ T cells, isolated from spleens of T cell-specific NRK1KO mice (NRK1Flox+/CD4Cre+) or littermate Cre-recombinase negative controls (NRK1Flox+/CD4Cre-) were cultured for 6 days under (B-D) Th17 or (E-G) Th0/Th2 polarising conditions and assessed for (**B-C**) intracellular IL-17 and RORγt abundance (E) intracellular GATA3 and IFN-γ abundance and (**D,G**) viability by flow cytometry (summarised for C,D: n=13 NRK1Flox+/CD4Cre- and n=16 NRK1Flox+/CD4Cre+ animals. F-G n=7 individual animals per group). p values were calculated by (C-D) unpaired t test. * $p < 0.05$. (C) p=0.0194, p=0.0215. Source data are provided as a Source Data file.

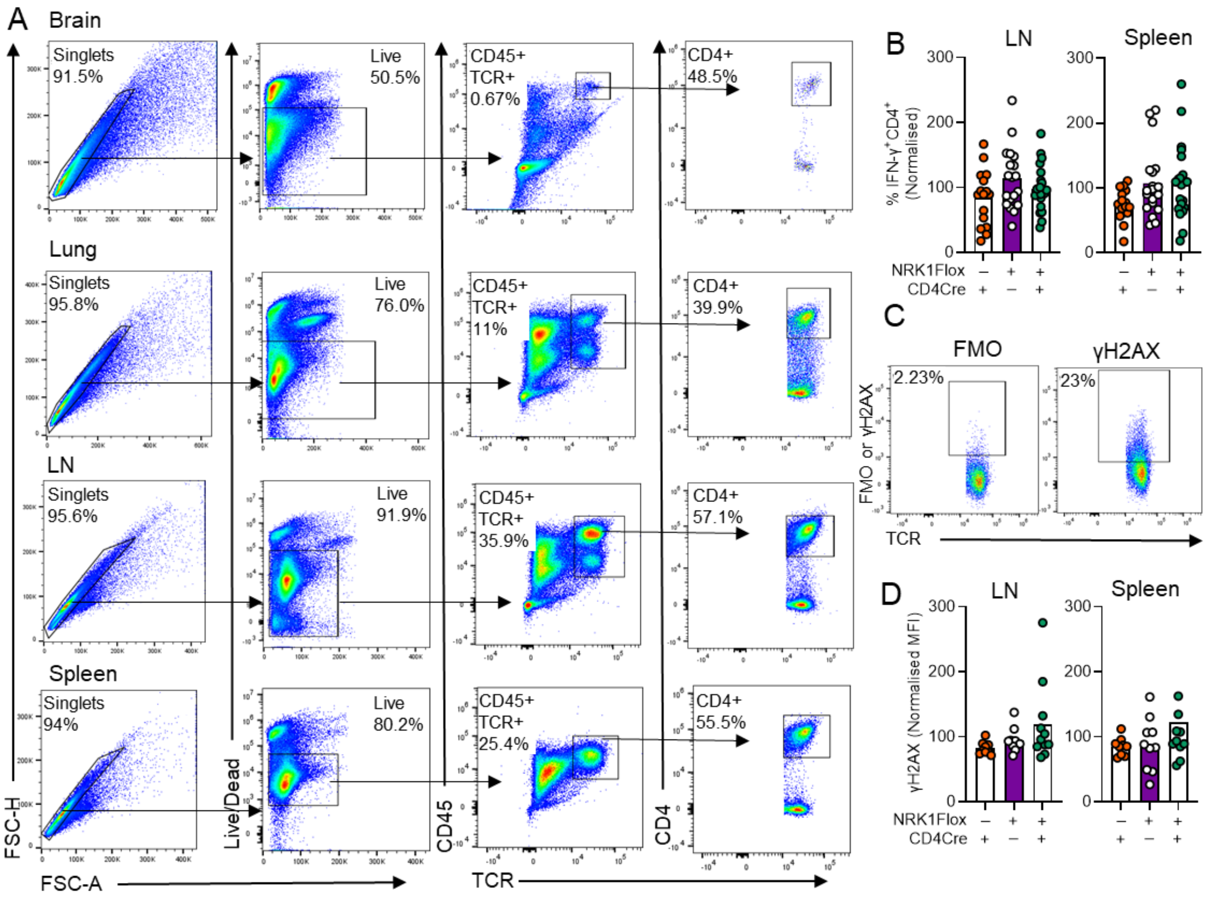

Supplementary Figure 9: (Related to Figure 8)

(**A-D**) Cre-recombinase control (NRK1Flox-/CD4Cre+), NRK1Flox+/CD4Cre- or NRK1Flox+/CD4Cre+ mice were infected intranasally with *C. neoformans* and indicated tissues analysed ((**A**) Example of gating strategy) at day 14 for (**B**) frequency of IFN-γ^{+} CD4^{+} T cells in Figure 8A and Supp Fig 9B and (**C-D**) DNA damage within CD4^{+} T cells (γH2AX abundance) by flow cytometry in Figure 8B and Supp Fig 9D (C, example of fluorescence minus one (FMO) staining (summarised for B: n=16 NRK1Flox-/CD4Cre+, n=17 NRK1Flox+/CD4Cre- and n=20 NRK1Flox+/CD4Cre+; D: n=7 NRK1Flox-/CD4Cre+, n=10 NRK1Flox+/CD4Cre- and n=11 NRK1Flox+/CD4Cre+ animals). Data were compiled from 5 independent experiments). Where normalised, data are expressed as a percentage of the average (mean) value across all samples analysed together for a batch of mice, of equivalent numbers of each genotype.

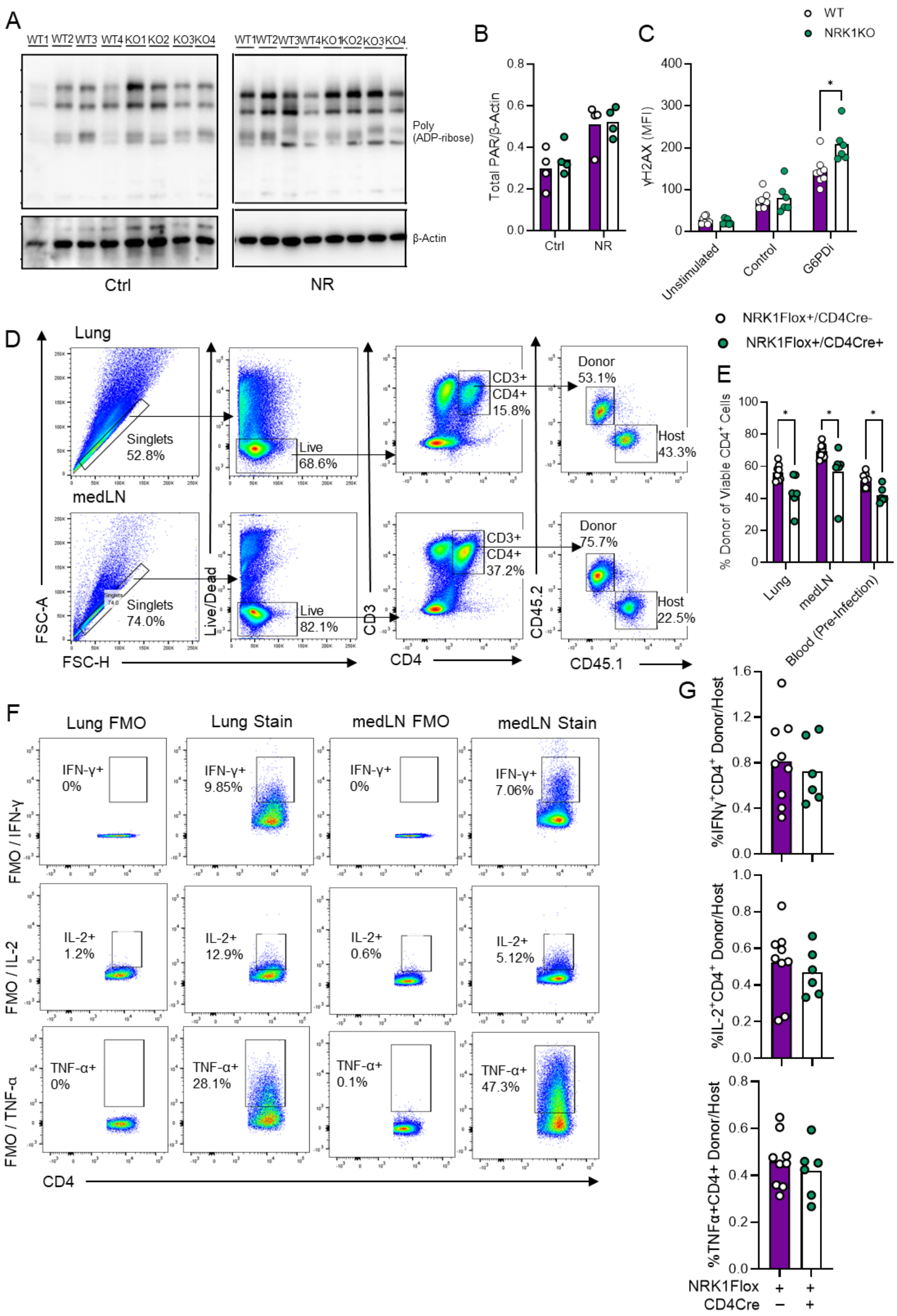

Supplementary Figure 10: (Related to Figure 8)

(**A-B**) Murine CD4$^+$ T cells isolated from spleens of littermate WT or NRK1KO animals as indicated, cultured ± stimulation via CD3/CD28 and ± NR (0.5mM) were assessed for protein poly (ADP-ribose) (PAR) modification by western blot with PAR-binding reagent (A) example blots for control and NR-treated WT and NRK1KO cells and (B) data summarised relative to β-actin for n=4 individual animals per group). (**C**) Murine CD4$^+$ T cells isolated from spleens of littermate WT or NRK1KO animals as indicated, cultured ± stimulation via CD3/CD28 and ± G6PD inhibitor (G6PDi, 50µg/ml) were assessed for DNA damage within CD4$^+$ T cells (γH2AX abundance) by flow cytometry (summarised for n=6 individual animals per group). (**D-G**) Murine bone marrow chimaera recipients of NRK1Flox+/CD4Cre- or NRK1Flox+/CD4Cre+ haematopoietic cells as indicated were infected intranasally with *Influenza A* and indicated tissues analysed at day 9 (D) example of gating strategy for Figure 8F-G and Supp Fig 10G, (E) frequency of viable donor CD4+ T cells summarised for indicated tissues (n= 9 NRK1Flox+/CD4Cre-, n=6 NRK1Flox+/CD4Cre+ animals), (F) example fluorescence minus one (FMO) controls for indicated cytokine staining and indicated tissues (medLN = mediastinal lymph node), (G) frequency of cytokine-expressing CD4$^+$ T cells within lung CD4$^+$ T cells (expressed as a ratio within donor (CD45.2) / host (CD45.1) cells, n= 9 NRK1Flox+/CD4Cre-, n=6 NRK1Flox+/CD4Cre+ animals). p values were assessed by (B-C,E) two-way ANOVA and Holm-Sidak's post-hoc test (G) unpaired t test. * $p < 0.05$. (E) P=0.0104, P=0.0104, P=0.0292. Source data are provided as a Source Data file.

Antibody	Use	Clone	Fluorophore	Supplier	Cat#	Conc.
**Human CD4⁺ T cell Analysis**						
Anti-human NRK1	WB<br>FACS	EPR11190	Unconjugated	abcam	Ab169548	1 in 1000<br>1 in 200
Anti-human CD25	FACS	BC96	BV605	Biolegend	302632	1 in 50
Anti-human CD69	FACS	FN50	APC	Biolegend	310910	1 in 50
Anti-human CD4	FACS	OKT4	FITC	Biolegend	317408	1 in 50
Anti-human CD8	FACS	SK1	BV510	Biolegend	344732	1 in 50
Anti-human IFN-gamma	FACS	B27	FITC	Biolegend	506504	1 in 50
Anti-human TNF-alpha	FACS	MAb11	PE	Biolegend	502909	1 in 150
Anti-human CD3	Cell Culture	OKT3	N/A	Biolegend	317326	1µg/ml
Anti-human CD28	Cell Culture	CD28.2	N/A	Biolegend	302943	5µg/ml
Anti-human CD45	Confocal	HI30	Unconjugated	Proteintech	CL647-65109	1 in 200
Anti-human HSP60	Confocal	4B9/89	Unconjugated	ThermoFisher	MA3-012	1 in 50
**Murine CD4⁺ T cell Analysis**						
Anti-mouse CD3	Cell Culture	145-2C11	N/A	Biolegend	100340	1µg/ml
Anti-mouse CD28	Cell Culture	37.51	N/A	Biolegend	102121	5µg/ml
Anti-mouse IFN-γ	FACS	XMG1.2	FITC	Biolegend	505806	1 in 100
Anti-mouse TNF-α	FACS	MP6-XT22	PE	Biolegend	506306	1 in 200
Anti-mouse IL-2	FACS	JES6-5H4	BV421	Biolegend	503826	1 in 100
Anti-mouse CD4	FACS	GK1.5	APC	Biolegend	100412	1 in 200
Anti-mouse CD4	FACS	GK1.5	AF-700	Biolegend	100429	1 in 200
Anti-mouse CD8	FACS	53-6.7	PE-CY7	Biolegend	100722	1 in 200
Anti-mouse CD19	FACS	6D5	PE	Biolegend	115508	1 in 200
Anti-mouse CD25	FACS	PC61	AF-700	Biolegend	102024	1 in 200
Anti-mouse CD45	FACS	30-F11	BV605	Biolegend	103139	1 in 200
Anti-mouse CD45.1	FACS	A20	Percp5.5	Biolegend	110728	1 in 200
Anti-mouse CD45.2	FACS	104	BV421	Biolegend	109832	1 in 200
Anti-mouse CD69	FACS	H1.2F3	BV605	Biolegend	104529	1 in 200

Anti-mouse PD-1	FACS	29F.1A12	PE	Biolegend	135205	1 in 200
Anti-mouse TCRβ	FACS	H57-597	PerCP/Cy5.5	Biolegend	109228	1 in 200
Anti-mouse FOXP3	FACS	MF-14	BV421	Biolegend	126419	1 in 50
Anti-mouse CD62L	FACS	MEL-14	APC/CY7	Biolegend	104427	1 in 200
Anti-mouse IL-17	FACS	TC11-18H10.1	PE-Cy7	Biolegend	506921	1 in 200
Anti-mouse RORγt	FACS	B2D	PE	ebioscience	12698180	1 in 200
Anti- mouse GATA3	FACS	W19195B	PE	Biolegend	386903	1 in 200
Anti-puromycin	FACS	2A4	AF488	Biolegend	381606	1 in 100
Anti-γH2AX	FACS	2F3	FITC	Biolegend	613404	1 in 100
Poly (ADP-ribose) (PAR) detection reagent	WB	N/A	N/A	Merck	MABE1031	1 in 1000
**Reagents used for both human and murine $CD4^+$ T cell analysis**						
Fixable viability dye eFluor™ 780	FACS	N/A	APC-Cy7	Invitrogen	65-0865-14	1 in 1000
Zombie violet fixable viability kit	FACS	N/A	BV421	Biolegend	423114	1 in 500
NFAT1 XP(R) Rabbit mAb	FACS	D43B1	AF647	Cell Signalling Technology	14201S	1 in 50
Donkey anti-rabbit IgG (H+L)	FACS	Polyclonal	AF555	Invitrogen	A31572	1 in 500
Donkey anti-rabbit IgG (min. x-reactivity)	FACS	Polyclonal	BV421	Biolegend	406410	1 in 100

Table S1: Antibody Details

**Gene**	**Primer**	**Sequence**
Murine NRK1	Forward	TCATTGGAATTGGTGGTGTGAC
	Reverse	CAACAGGAAACTGCTGACATCAT
Murine NAMPT	Forward	GCAGAAGCCGAGTTCAACATC
	Reverse	TTTTCACGGCATTCAAAGTAGGA
Murine NMNAT1	Forward	TGGGGCCAATGAGAGCAAG
	Reverse	CAACCCTCTGACAGCGATGTT
Murine NNMNAT3	Forward	CCTGTGGTTCCTTCAACCCC
	Reverse	AGATGATGCCCTCAATCACCT

Table S2: Primer Details – mRNA transcript analysis

Gene/Region	Primer	Sequence
Murine *Nrk1*<br>Ch19: 18618741:18618813	Forward	AGTGCTCAAGGGGTTCCCATTTT
	Reverse	GACACCACATTCAGGCCACAAG
Murine *Nrk1*<br>Ch19: 18618391:18618558	Forward	ACAGAGCCCTGGAGATGCTT
	Reverse	AGAGCTTCCTGGGTTTTCCA
Murine *Il2ra*<br>(Jennings et al, STAR protocols, 2021, DOI:10.1016/j.xpro.2020.100284)	Forward	CAGGAGTTTCCTAAGCAACG
	Reverse	CTGTGTCTGTATGACCCACC
Tm1d recombined allele	Forward	AAGGCGCATAACGATACCAC
	Reverse	ACTGATGGCGAGCTCAGACC

Table S3: Primer Details – genomic DNA analysis

Supplemental Methods

Human CD4⁺ T cell isolation and culture

Total CD4⁺ T cells were isolated from human peripheral blood by density-gradient centrifugation followed by positive selection using human CD4 Microbeads (Miltenyi, Cat# 130-045-101), purity was typically >95%. Cells were cultured at a density of 1 x 10^6 cells/ml in (unless otherwise indicated) RPMI-1640 containing 10% foetal calf serum (FCS) (Sigma Aldrich, Cat# F9665), 50 U/mL penicillin and 50 mg/mL streptomycin (Thermo Fisher Scientific, Cat# 15140122), and 50 IU/mL rIL-2 (PeproTech, Cat# 200-02) (RPMI/FCS). Cells were activated with 12 µl/ml ImmunoCult Human CD3/CD28 T Cell Activator (STEMCell, Cat# 10991) or, where indicated, plate bound anti-CD3 and/or anti-CD28 antibodies (**Table S1**).

Murine splenocyte and CD4⁺ T cell isolation and culture

Murine splenocytes, thymocytes and lymph node mononuclear cells were isolated by manual disruption of tissue into a paste followed by washing twice with RPMI/FCS and filtration through a 70µM filter. Red blood cells were lysed in RB lysis buffer (Invitrogen, Cat# 00-4333-57)) on ice for 2 minutes before washing in PBS. Total CD4⁺ T cells were isolated from murine splenocytes by positive selection using mouse CD4 Microbeads (Miltenyi, Cat# 130-117-043), purity was typically >95%. Cells were cultured at a density of 1 x 10^6 cells/ml in (unless otherwise indicated) RPMI/FCS supplemented with 50µM β-mercaptoethanol (Gibco, Cat# 21985023). Cells were activated with plate bound anti-CD3 and anti-CD28 antibodies (**Table S1**). CD19⁺ B cells were isolated from splenocytes by flow cytometry cell sorting (**Table S1**) and stimulated for 24 hours with 50ng/ml LPS (Sigma-Aldrich, Cat#437620).

Additions to cell culture

Additions to human and/or murine cell cultures, where indicated, included ERKi (PD0325901, Cambridge Biosciences, Cat# SM26-2), SRCi (PP2, Sigma-Aldrich, Cat# P0042), Pi3Ki (Ly294002, Promega, Cat# V1201), Akti (abcam, Cat# ab142088), ciclosporin A (Cambridge Biosciences, Cat# SM43-50) nicotinamide riboside (NR, Sigma-Aldrich Cat# SMB00907), nicotinamide adenine mononucleotide (NAM, Sigma-Aldrich, Cat# N0636), FK866 (NAMPTi, Sigma-Aldrich, Cat#F8557), G6PD inhibitor (G6PDi, Sigma-Aldrich, Cat# SML2980). Vehicle controls were added equivalent to the highest volume of additions. For Th1, Th17 and Th2 polarisation, cells were stimulated via CD3/CD28 as above, for 6 days in presence of Th1: IL-12 (10ng/ml; Biolegend, Cat# 575402); Th17: IL-6 (25ng/ml; Biolegend Cat# 575706),

IL-1β (10ng/ml; Biolegend, Cat# 575104), TGFβ (2ng/ml; Biolegend Cat# 580706), anti-IFN-γ (20ng/ml; Biolegend, QA20A28, Cat# 606853); Th2: IL-4 (10ng/ml; Biolegend Cat# 574302), anti-IFN-γ (10ug/ml).

Western Blot

Cell lysates were prepared in RIPA buffer (Thermo Fisher Scientific, Cat# 89900) supplemented with protease and phosphatase inhibitor (Sigma-Aldrich, Cat# PPC1010). Protein concentrations determined with a BCA protein assay kit (Thermo Fisher Scientific, Cat# 23225). Whole-cell lysates were resolved by 10% SDS-PAGE and were transferred onto nitrocellulose membranes. The membranes were then incubated with primary antibody or poly (ADP-ribose) (PAR) detection reagent (**Table S1**), secondary goat anti-rabbit HRP (Dako, Cat# P0448, 1:5000) and the HRP-ECL system (Bio-Rad Clarity Western ECL Substrate Cat# 170-5061) used for band detection. Please refer to Source Data files for uncropped images of NRK1 and beta-actin blots.

qPCR

Relative abundance of mRNAs of interest were quantified by real-time RT-PCR (qPCR). The mRNA was extracted with NucleoSpin RNA Mini Kit (Machery-Nagel, Cat# 740955.5) from 4 x 10^6 cells and cDNA was transcribed with the Promega reverse transcription reagents (PCR mix, Cat# U110A; Oligo dt primer, Cat# C110A; Rev transcriptase, Cat# M170A; RNasin, Cat# N261A) according to the manufacturer's instructions. SYBR green primers were used for qPCR analysis. See **Table S2** for further details. For genomic DNA analysis, DNA was extracted from 1 x 10^6 indicated cell types using PureLink Genomic DNA mini Kit (Thermo Fisher). SYBR green primers were used (**Table S3**).

End-stage PCR

PCR was performed using the One-taq hot-start PCR mix (New England Biolabs, MO481S) according to manufacturer's instructions. 35 cycles of PCR amplification were performed (94C 15sec, 58C 30 sec, 68C 75 sec) with a final extension stage of 68C for 10 minutes. PCR products were resolved on a 1% agarose gel with a 100 bp ladder.

NAD/H and NADP/H measurement

Total NAD/H or NADP/H were measured within whole cell lysates or cytoplasmic fractions of human and murine $CD4^+$ T cells using Promega Bioluminescent assays (NAD/NADH-Glo™ (Promega, Cat# G9071) and NADP/NADPH-Glo™ Promega, Cat# G9081) to manufacturer's instructions, analysed with a CLARIOstar Plus plate reader (BMG Labtech).

Flow cytometry analysis of surface and intracellular protein expression

For analysis of cell surface protein expression, human and murine cells were stained in buffer containing 1x PBS and 2% FCS (FACS Buffer) with specific monoclonal antibodies for 30 minutes at 4°C then washed twice with FACS buffer prior to analysis. Details of surface antibodies used are provided in **Table S1**. Cells were stained with Fixable Viability Dye (**Table S1**) during staining to assess cell viability. For assessment of intracellular protein expression, cells were stained for viability and cell surface protein expression as before, washed twice with FACS buffer, then fixed using FoxP3 fixation/permeabilization solution (eBioscience, Cat# 005523-00) for 20 minutes at 4°C. Cells were washed once with FoxP3 permeabilization buffer and incubated with antibodies for 30-60 minutes (**Table S1**). After washing a further two times cells were either analysed or incubated with secondary donkey anti-Rabbit antibody IgG1 (**Table S1**) for 20 minutes at room temperature. Cells were then again washed twice before analysis. Data were analysed using TreeStar FlowJo v10.

Flow cytometry analysis of intracellular cytokine abundance

For analysis of intracellular cytokine abundance, cells were first activated for 4 hours with Cell Activation Cocktail with Brefeldin A (BioLegend Cat# 423,304) prior to fixation, permeabilization and intracellular staining as described above. Antibody details are provided in **Table S1**.

ELISA

Cell culture supernatants from human or murine $CD4^+$ T cell cultures were harvested and stored at -20°C for analysis by ELISA. Human IFN-γ concentration was measured using anti-IFN-γ capture (Bio-Rad, Clone AbD00676, Cat# HCA043) and biotinylated detection (Bio-Rad, Clone 2503 Cat# HCA044P) antibodies, recombinant IFN-γ standard (Bio-Rad, Cat# PHP050), streptavidin-HRP (Sigma-Aldrich, Cat# E2866) and TMB substrate (BD Biosciences, Cat# 555214). Human TNF-α concentration was measured using TNF-α antibody pair and ELISA buffer kit (Invitrogen, Cat# CHC1753 and Cat# CNB0011). Murine IFN-γ and TNF-α were quantified using ELISA MAXTM Deluxe Set Mouse IFN-γ (Biolegend, Cat# 430804) and ELISA MAXTM Deluxe Set TNF-α (Biolegend, Cat# 430904) to manufacturer's instructions.

Extracellular flux analysis

Oxygen consumption rate (OCR, pmol/minute) and extracellular acidification rate (ECAR, mpH/minute) were analysed using the Seahorse XFe96 metabolic extracellular flux analyser (Agilent). Murine $CD4^+$ T cells were cultured as described for 48 hours before analysis. Cells were resuspended in Seahorse XF RPMI (Agilent, Cat# 103576-100) and

plated onto Seahorse cell plates (2.5×10^5 cells per well) coated with poly-d-lysine (Gibco, Cat# 10317081) to promote T cell adherence. Metabolic profiles were created by the addition of oligomycin (1 µM), Bam-15 (3 µM), and rotenone/antimycin A (both 2 µM; all given as final concentrations, all from Sigma-Aldrich). Basal OCR was calculated as the mean of the initial 3 measurements minus the mean of the 3 measurements after rotenone/antimycin A injection. ATP-coupled OCR was calculated as Basal OCR minus the mean of the 3 measurements after oligomycin injection. Maximal OCR was calculated as the mean of the 3 measurements after Bam-15 injection minus the mean of the 3 measurements after rotenone/antimycin A injection. Basal ECAR was calculated as the mean of the 3 initial measurements and maximal ECAR as the mean of the 3 measurements after rotenone/antimycin A injection. Analysis was performed using Agilent Wave Software.

SCENITH (single-cell energetic metabolism by profiling translation inhibition) Assay

To undertake this assay[20], murine $CD4^+$ T cells were incubated in RPMI/FCS in presence of oligomycin (1 µM), 2-deoxyglucose (2-DG, 10mM (Sigma-Aldrich, Cat# D6134), both compounds, or vehicle control (DMSO) for 15 minutes at 37̊C, 5% CO_2, prior to addition of puromycin (50µg/ml, Sigma-Aldrich Cat# P4512) and incubation for another 15 minutes. Cells were then immediately fixed with FoxP3 fixation/permeabilization solution. After permeabilization with FoxP3 permeabilization buffer, cells were stained with anti-puromycin-Alexa fluor 647 (**Table S1**) for 20 minutes at room temperature, washed and analysed by flow cytometry. Mitochondrial dependence was calculated as "100 x (puromycin MFI of vehicle control – puromycin MFI of oligomycin-treated sample) / (puromycin MFI of oligomycin-treated sample – puromycin MFI of oligomycin + 2-DG-treated sample)". Glycolytic capacity was calculated as "100 – mitochondrial dependence".

Stable isotope based metabolic tracing – universally-labelled substrates, GC-MS analysis

Murine $CD4^+$ T cells (4×10^6 per condition) were cultured as described for 48 hours at 37°C, 5% CO_2 in SILAC RPMI-1640 Flex Media (Gibco, Cat# A2494201) supplemented with 10% FCS, rIL-2 (50 IU/ml), L-Lysine (4 µg/ml; Sigma-Aldrich, Cat# L5501), L-Arginine (20 µg/ml; Sigma-Aldrich, Cat# A8094) and either 10mM universally-labelled (U)-$^{13}C_6$ glucose (CK Isotopes, Cat# CLM-1396-1) and 2mM unlabelled glutamine (Sigma-Aldrich, Cat# G7513) or 2mM U-$^{13}C_5$ glutamine (CK Isotopes, Cat# CNLM-1275) and 10 mM unlabelled glucose (Fisher Scientific, Cat# 10141520 Cells were then washed with ice-cold 0.9% saline solution and were extracted in 5:2:5 pre-chilled HPLC-grade methanol, HPLC-grade water (containing 1.75 µg/mL D6-glutaric acid) and chloroform. The extracts were shaken at 1400 rpm for 15

minutes at 4°C and centrifuged at 12,000 g for 15 minutes at 4°C. The upper aqueous phase was collected and evaporated under vacuum. Metabolite derivatization was performed using a two-stage derivatization protocol. Dried polar metabolites were dissolved in 20 µL of 2% methoxyamine hydrochloride in pyridine (Thermo Fisher Scientific, Cat# 25104) at 60°C for 60 minutes, followed by 30µl of N-tert-Butyldimethylsilyl-N-methyltrifluoroacetamide with 1% tertbutyldimethylchlorosilane, incubated at 60°C for 60 minutes. GC-MS analysis was performed using an Agilent 6890GC equipped with a 30m Rxi-5ms (0.25 mm ID) capillary column. The GC was connected to an Agilent 5975C MS operating under electron impact ionization at 70 eV. The MS source was held at 230°C and the quadrupole at 150°C. The detector was operated in scan mode and 1 µL of derivatised sample was injected in splitless mode. Helium was used as a carrier gas at a flow rate of 1 mL/minute. The GC oven temperature was held at 100°C for 1 minute and increased to 160°C at a rate of 10°C/minute, increased to 200°C at 5°C/minute, and a final increase to 330°C at 10°/minute and held for 4 minutes. The run time for each sample was 32 minutes. For determination of the mass isotopomer distributions (MIDs), spectra were corrected for natural isotope abundance. Data processing was performed using MATLAB.

Stable isotope based metabolic tracing – 1,2-labelled glucose, LC-MS analysis

Murine $CD4^+$ T cells (4 x 10^6 per condition) were cultured as described for 48 hours in SILAC RPMI-1640 Flex Media (Gibco, Cat# A2494201) supplemented with 10% FCS, rIL-2 (50 IU/ml), L-Lysine (4 µg/ml; Sigma-Aldrich, Cat# L5501), L-Arginine (20 µg/ml; Sigma-Aldrich, Cat# A8094), 10mM 1,2-$^{13}C_2$ glucose (CK Isotopes, Cat# CLM-504-0.25) and 2mM unlabelled glutamine. Cells were washed with ice-cold 0.9% saline solution metabolites were extracted with 100 µL of ice-cold extraction buffer (40% methanol (Biosolve, BIO-13687802), 40% acetonitrile (Biosolve, BIO-01204102), 20% water (Biosolve, BIO-23214102-1), 15 µM glutaric acid internal standard and 0.5% formic acid (Biosolve, BIO-069141A8)) for 5 minutes on ice. 8.8 µL of 15% ammonium bicarbonate (Supelco, 5.33005) solution was added to neutralise and samples were left on dry ice for a further 15 minutes. Samples were thawed on ice and then centrifuged at 20 G at 4°C for 5 minutes. Supernatants were collected for LC-MS analysis. LCMS analysis was performed on an Agilent LCMS QToF 6546 using a Waters Premier BEH Z-HILIC VanGuard Fit column (1.7 µm, 2.1 mm x 150 mm) with the following mobile phases: Mobile phase A: 20 mM Ammonium hydrogen carbonate (LiChropur, Supelco) in LC-MS grade water (Ultra CHROMASOLV, Honeywell Riedel-de Haën) with 0.1% ammonium hydroxide (Alfa Aesar, Thermo Scientific) and 5 µM InfinityLab deactivator additive (Agilent Technologies). Mobile phase B: 90% UHPLC grade acetonitrile (BioSolv, Greyhound Chemicals), 10% LC-MS grade water (Ultra CHROMASOLV, Honeywell

Riedel-de Haën) with 5 uM InfinityLab deactivator additive (Agilent Technologies). 5 µl of sample was injected and the chromatographic separation was achieved with a gradient run with a constant flow rate of 0.2 mL/minute and the following program: T = 0 min, 10% A, 90% B; T = 2 min, 10% A, 90% B; T = 18 min, 35% A, 65% B; T = 22 min, 65% A, 35% B; T = 22.1 min, 85% A, 15 % B; T = 25 min, 85% A, 15% B; T = 25.1 min, 10% A, 90% B; T = 30 min, 10% A, 90% B. Full scan data was acquired between m/z 50 – 1050 at 1 Hz whilst using online mass correction. Analyte ionisation was achieved via ESI (negative polarity) with the following parameters: VCap: 2000 V, Nozzle Voltage: 500 V, gas temperature: 225 °C, drying gas: 8 l/min, nebulizer: 30 psi, sheath gas temp: 300 °C, sheath gas flow 12 l/min. Data extraction was performed using Agilent Profinder 10.0. Data was normalised to the internal standard (D6-Glutaric acid) peak area using an in-house R script.

GSH/GSSG measurement

Total GSH/GSSG ratios were measured within whole cell lysates of human and murine $CD4^+$ T cells using a bioluminescent assay (Promega, Cat# V6611) according to manufacturer's instructions and analysed using CLARIOstar Plus (BMG Labtech).

Cellular reactive oxygen species (ROS) measurement

Cells were stained with 2′,7′-Dichlorofluorescin Diacetate (DCFDA, Sigma-Aldrich Cat# 287810) to assess for differences in ROS. Cells (0.2×10^6) were incubated in RPMI (without FCS) with 20µM DCFDA for 20 min at 37°C and 5% CO_2 before undergoing washing and analysis by flow cytometry.

Nuclei isolation for NFAT analysis

Sucrose buffer A was prepared with 10 mM HEPES, 8 mM $MgCl_2$, 320 mM sucrose (Sigma-Aldrich, catalogue #S8501), 0.1% Triton-X 100 (Sigma-Aldrich, Triton™ X-100, catalogue #X-100), protease and phosphatase inhibitor (Sigma-Aldrich, Protease and Phosphatase Inhibitor Cocktail, catalogue #PPC1010), and diluted with distilled H_2O. Sucrose buffer B was prepared as for 'sucrose buffer A' but without Triton-X 100. $CD4^+$ T cells were activated as indicated prior to washing and incubation for 15 minutes on ice with 'sucrose buffer A'. Cells were then centrifuged at 2000 g for 5 minutes at 4℃ before 2 washes with 'sucrose buffer B' at the same centrifuge settings. Cells were fixed using FoxP3 fixation/permeabilization solution for 30 minutes on ice in the dark. After centrifugation and removal of the supernatant, the nuclei pellets were washed once with FACS buffer and $MgCl_2$ at 1000 g for 5 minutes at 4℃, and once with FOXP3 permeabilization buffer and $MgCl_2$ at the same centrifuge settings. Nuclei were stained with

fluorescent-labelled antibodies (**Table S1**) in FOXP3 permeabilization buffer and $MgCl_2$ for 1 hour at 4°C. The nuclei pellets were washed twice in FACS buffer and $MgCl_2$ prior to running on the flow cytometer.

Confocal microscopy

Human $CD4^+$ T cells, previously cultured as indicated, were allowed to attach by gravity for 20 minutes onto culture slides (BD Bioscience) coated with poly-d-lysine. Attached cells were fixed with 4% paraformaldehyde and permeabilized with 0.3% Triton X-100. Cells were stained with antibodies detailed in **Table S1** and mounted in ProLong™ Gold Antifade with DAPI (Thermofisher, catalogue #P36941). Images were then collected using a Zeiss LSM 880 with Airyscan Fast. For analysis of the cytoplasmic localization of NRK1, the nuclear stain was used as the mask, and fluorescence intensity of cytoplasmic NRK1 (outside nuclear mask) was quantified. All images were processed with ImageJ software (US National Institutes of Health).

NAD biosensor preparation and use

DNA plasmid vectors containing NAD+ biosensors were expanded from Agar stabs according to protocols set out by Addgene (Addgene, Cytosolic NAD+ biosensor Cat #186787, Cytoplasmic cpVenus control Cat #186788, Nuclear NAD+ biosensor Cat #186789, Nuclear cpVenus control Cat #186790, Mitochondrial NAD+ biosensor Cat #186791, Mitochondrial cpVenus Cat #186792). A sterile inoculating loop was used to acquire bacteria from the agar stab and streak onto agar plates containing Ampicillin 100μg/ml (Sigma-Aldrich Cat #59349) which were incubated at 37 °C overnight. Following this, under sterile conditions, single colonies were selected for each plasmid vector and inoculated into 5ml of LB broth (ThermoFisher Cat #12780052) containing 100μg/ml Ampicillin then incubated in a shaking incubator for 12-18 hours at 37 °C. After incubating, glycerol stocks for each plasmid were created by adding 500 µL of this liquid culture into a solution of 50% glycerol and the resulting glycerol stocks were stored at -80°C. The vectors were later expanded from Glycerol stocks and the plasmids were extracted using to ZymoPURE™ Plasmid Midiprep Kit (Cat# D4200) according to manufacturer's protocol. The resulting plasmids were stored at -20°C.

Electroporation was performed 24 hours after T cell stimulation. In brief, 1×10^6 prewashed $CD4^+$ T cells were resuspended in 100 µl electroporation buffer (Geneflow, Cat#E7-0516) including 5 µg DNA plasmid vectors. Cells were then moved into electroporation cuvettes and electroporated with (Lonza 2b Nucleofector), using the stimulated human T cell programme. Cells were immediately supplemented with prewarmed medium (RPMI, 20% FCS) and transferred out of the electroporation cuvettes. After 16 hours, cells were washed and resuspended in

RPMI/FCS and distributed into a 96 well plate to then be treated with indicated compounds for 6 hours and then analysed by flow cytometry. Data analysis was performed as described[25,26] with NAD-dependent changes in biosensor fluorescence at 530nm (excitation 488nm) normalised to NAD-insensitive fluorescence at 510nm (excitation 405nm). A second normalisation step expressed alterations in this ratio relative to those of control cpVenus protein, which do not have a NAD+ binding pocket, meaning fluorescence is not affected by NAD+.

Data analysis was performed as described[25,26] with NAD-sensitive biosensor fluorescence at 530nm (excitation 488nm) divided by NAD-insensitive fluorescence at 510nm (excitation 405nm). This was performed in parallel for cells expressing control cpVenus proteins, which do not have a NAD+ binding pocket, meaning fluorescence at neither wavelength is affected by NAD+. A ratio was then calculated by dividing biosensor values by cpVenus values. Finally, to obtain "ratio of ratios", identifying effects of treatment, the ratio value acquired from the cells which were treated were divided by the ratio value of the cells which were not treated.

Digitonin treatment for cytoplasmic NAD(P)/H quantification

Cytoplasmic fractions from human and murine $CD4^+$ T cells (1 x 10^6) were extracted by washing cells with 0.9% saline, resuspending in digitonin solution (Sigma Aldrich, Cat#141, 100µg/ml in 0.9% saline) for 3 minutes at room temperature, vortexing, centrifugation (400g, 5 minutes) and collection of resulting supernatant containing the cytoplasm.

***Cryptococcus neoformans* infection model**

C. neoformans strain H99 was used in this study, as in[42]. Yeast was routinely grown in YPD broth (2% peptone (Fisher Scientific), 2% glucose (Fisher Scientific), and 1% yeast extract (Sigma-Aldrich) at 30 °C for 24 hours at 200 rpm. For infections, yeast cells were washed twice in sterile PBS, counted using haemocytometer, and $2x10^5$ yeast administered intranasally in 25µl, under isoflurane anaesthesia. For analysis of brain and lung fungal burdens, animals were euthanized and organs weighed, homogenized in PBS, and serially diluted before plating onto YPD agar supplemented with Penicillin/Streptomycin. Colonies were counted after incubation at 30 °C for 48 hours.

Leukocytes were isolated from brains that had been aseptically removed and stored in ice-cold FACS buffer by homogenisation into a paste and density-gradient centrifugation. The suspension was resuspended in 10 mL 30% Percoll (GE Healthcare), and underlaid with 1.5 mL of 70% Percoll. Gradients were centrifuged at 1000 g for 30 minutes at 4 °C with the brake off. Leukocytes at the interphase were collected and washed in FACS buffer.

Leukocytes were isolated from lungs that had been aseptically removed, finely minced with a scalpel and stored in ice-cold digest buffer (RPMI, 10% FCS, 1% penicillin-streptomycin, 1mg/ml collagenase D (Fisher Scientific, Cat# 10780004), 1 mg/ml Dispase II (Sigma-Aldrich, Cat# D4693) and 40μg/ml DNase I (Sigma-Aldrich, Cat# 10104159001)) prior to digest at 37°C for 40 minutes with intermittent shaking. Digested lung tissue was passed through a 100μM filter and centrifuged at 400g for 5 minutes. Red blood cells were lysed in RBL lysis buffer (Invitrogen, Cat# 00-4333-57)) on ice for 2 minutes before washing in PBS. Sample was passed through a 40μM filter and leukocytes were collected and washed in FACS buffer.

Leukocytes were analysed from spleens and lymph nodes as described above. Brain, lung, spleen and lymph node leukocytes were then stained for flow cytometry analysis as described above.

Influenza A infection model

Bone marrow chimaeras were generated by irradiating mice twice with 4.25Gy 3 hours apart and then transplanting 2×10^6 whole bone marrow cells from donor mice intravenously. Peripheral blood reconstitution was assessed by flow cytometry 6 weeks after transplantation.

Mice were briefly anesthetized using inhaled isoflurane and infected with 50,000 plaque-forming units of Influenza A virus (IAV) X-31(H3N2) strain in 50 μL of phosphate-buffered saline (PBS) i.n. IAV was prepared and titered in Madin-Darby Canine Kidney cells (MDCK). Infected mice were weighed daily from day 4 post-infection. Any animals that lost more than 20% of their starting weight were humanely euthanized.

LNs were collected in PBS; split open using forceps; and placed in 1 ml of RPMI 1640 containing deoxyribonuclease I (DNase) (200 μg/ml) (Roche), 100 U of collagenase I (Worthington Biochemical), and 500 U of collagenase IV (Worthington Biochemical); and incubated for 15 min at 37°C. Samples were triturated and incubated for a further 15 min at 37°C. Samples were washed with RPMI 1640 containing 10% FBS and filtered through a 70-μm Nytex filter. Cells were then stained for flow cytometry. Lungs were collected in PBS and transferred into gentleMACs tubes containing 3 ml of dispase (10 μg/ml; Gibco) and DNase I (200 μg/ml; Roche) in 3 ml of RPMI. Tissue was dissociated using the gentleMACS (Miltenyi) following the manufacturer's instructions. A total of 25 ml of FACS buffer (2% FBS and 0.05% NaN_3 in PBS) was added to samples, and this was then filtered through a 70-μm Nytex filter. Samples were incubated in red blood cell lysis buffer (500 ml of dH2O, 0.5 g of $KHCO_3$, 4 g of NH_4Cl, 5 ml of 10 nM EDTA, and 25 ml of FBS) for 5 min at room temperature (RT), then washed with FACS buffer, and stained for flow cytometry.
