# Supplementary material for: Cytoplasmic NAD/H synthesis via NRK1 regulates inflammatory capacity and promotes survival of CD4+ T cells
Source: Nat Commun. 2026 Feb 4;17:2349. doi: 10.1038/s41467-026-68863-w (PMC12979809; doi:10.1038/s41467-026-68863-w)
Supplement: Supplementary file 2 — Reporting Summary [file 41467_2026_68863_MOESM2_ESM.pdf]

nature portfolio

Corresponding author(s): Sarah Dimeloe

Last updated by author(s): Dec 15, 2025

Reporting Summary

Nature Portfolio wishes to improve the reproducibility of the work that we publish. This form provides structure for consistency and transparency in reporting. For further information on Nature Portfolio policies, see our Editorial Policies and the Editorial Policy Checklist.

Statistics

For all statistical analyses, confirm that the following items are present in the figure legend, table legend, main text, or Methods section.

n/a	Confirmed	
☐	☒	The exact sample size (*n*) for each experimental group/condition, given as a discrete number and unit of measurement
☐	☒	A statement on whether measurements were taken from distinct samples or whether the same sample was measured repeatedly
☐	☒	The statistical test(s) used AND whether they are one- or two-sided<br>*Only common tests should be described solely by name; describe more complex techniques in the Methods section.*
☒	☐	A description of all covariates tested
☐	☒	A description of any assumptions or corrections, such as tests of normality and adjustment for multiple comparisons
☐	☒	A full description of the statistical parameters including central tendency (e.g. means) or other basic estimates (e.g. regression coefficient) AND variation (e.g. standard deviation) or associated estimates of uncertainty (e.g. confidence intervals)
☐	☒	For null hypothesis testing, the test statistic (e.g. *F*, *t*, *r*) with confidence intervals, effect sizes, degrees of freedom and *P* value noted<br>*Give P values as exact values whenever suitable.*
☒	☐	For Bayesian analysis, information on the choice of priors and Markov chain Monte Carlo settings
☒	☐	For hierarchical and complex designs, identification of the appropriate level for tests and full reporting of outcomes
☐	☒	Estimates of effect sizes (e.g. Cohen's *d*, Pearson's *r*), indicating how they were calculated

Our web collection on statistics for biologists contains articles on many of the points above.

Software and code

Policy information about availability of computer code

Data collection	BD FACSDiva V9.0, BMG Labtech Voyager V2410, BMG Labtech Mars V2.10, Agilent WAVE v2.6, Thermo Fisher QuantStudio v6, Agilent Profinder 10.0,
Data analysis	Graphpad Prism V9 and 10, FlowJo V9 and V10, Microsoft Excel 365, MATLAB, Image J.

For manuscripts utilizing custom algorithms or software that are central to the research but not yet described in published literature, software must be made available to editors and reviewers. We strongly encourage code deposition in a community repository (e.g. GitHub). See the Nature Portfolio guidelines for submitting code & software for further information.

Data

Policy information about availability of data

All manuscripts must include a data availability statement. This statement should provide the following information, where applicable:

- Accession codes, unique identifiers, or web links for publicly available datasets
- A description of any restrictions on data availability
- For clinical datasets or third party data, please ensure that the statement adheres to our policy

The mass spectrometry data generated in this study have been deposited in the MassIVE database under accession codes MSV000100190 (GC-MS) and MSV000100189 (LC-MS) https://massive.ucsd.edu. All other data are available upon reasonable request to the corresponding author. Source data are provided as a Source Data file.

Research involving human participants, their data, or biological material

Policy information about studies with human participants or human data. See also policy information about sex, gender (identity/presentation), and sexual orientation and race, ethnicity and racism.

Reporting on sex and gender	Human immune cells were isolated from fully anonymised leukocyte cones provided by the National Health Service Blood and Transplant Service, without information on donor biological sex. However both sexes have equal opportunity to donote blood via this service. Experiments with immune cells from mice, or in vivo experiments performed in mice, always involved both sexes within each litter. Our findings are therefore applicable to both sexes.
Reporting on race, ethnicity, or other socially relevant groupings	Human immune cells were isolated from fully anonymised leukocyte cones provided by the National Health Service Blood and Transplant Service, without information on donor ethnicity. However, people of all ethnicities have equal opportunity to donote blood via this service.
Population characteristics	As described above, human immune cells were isolated from fully anonymised leukocyte cones provided by the National Health Service Blood and Transplant Service, without information on donor biological sex or ethnicity.
Recruitment	Donors are recruited by National Health Service Blood and Transplant Service.
Ethics oversight	Ethics approval is in place from the University of Birmingham STEM Ethics Committee, reference ERN_17-1743.

Note that full information on the approval of the study protocol must also be provided in the manuscript.

Field-specific reporting

Please select the one below that is the best fit for your research. If you are not sure, read the appropriate sections before making your selection.

[x] Life sciences [ ] Behavioural & social sciences [ ] Ecological, evolutionary & environmental sciences

For a reference copy of the document with all sections, see nature.com/documents/nr-reporting-summary-flat.pdf

Life sciences study design

All studies must disclose on these points even when the disclosure is negative.

Sample size	Sample sizes were based on power calculations from pilot experiments with n = 3-4 human donors or individual mice.
Data exclusions	Individual data points were removed if identified as outliers with the ROUT outlier test.
Replication	Experiments were repeated in multiple biological replicates - either individual human donors or mice. In addition, experiments were repeated on different dates/times (with independent donors or litters of mice) to control for inter-experimetnal technical variability.
Randomization	This is not relevant since treatments were either given to cells from the same human donor/mouse in vitro. For in vivo experiments, all mice were treated/infected and different genotypes were present within the same littler because of our heterozygote breeding strategy.
Blinding	Blinding was not possible for in vitro experiments, but was carried out for in vivo experiments, whereby mouse genotype was not known by the researcher who analysed mice/tissue samples.

Reporting for specific materials, systems and methods

We require information from authors about some types of materials, experimental systems and methods used in many studies. Here, indicate whether each material, system or method listed is relevant to your study. If you are not sure if a list item applies to your research, read the appropriate section before selecting a response.

Materials & experimental systems

n/a	Involved in the study	
[ ]	[x]	Antibodies
[x]	[ ]	Eukaryotic cell lines
[x]	[ ]	Palaeontology and archaeology
[ ]	[x]	Animals and other organisms
[x]	[ ]	Clinical data
[x]	[ ]	Dual use research of concern
[x]	[ ]	Plants

Methods

n/a	Involved in the study	
[x]	[ ]	ChIP-seq
[ ]	[x]	Flow cytometry
[x]	[ ]	MRI-based neuroimaging

Antibodies

Antibodies used

Antibody Use Clone Fluorophore Supplier Cat# Conc.
Human CD4+ T cell Analysis
Anti-human NRK1 WB/FACS EPR11190 Unconjugated abcam Ab169548 1 in 1000/1 in 200
Anti-human CD25 FACS BC96 BV605 Biolegend 302632 1 in 50
Anti-human CD69 FACS FN50 APC Biolegend 310910 1 in 50
Anti-human CD4 FACS OKT4 FITC Biolegend 317408 1 in 50
Anti-human CD8 FACS SK1 BV510 Biolegend 344732 1 in 50
Anti-human IFN-gamma FACS B27 FITC Biolegend 506504 1 in 50
Anti-human TNF-alpha FACS MAb11 PE Biolegend 502909 1 in 150
Anti-human CD3 Cell Culture OKT3 N/A Biolegend 317326 1μg/ml
Anti-human CD28 Cell Culture CD28.2 N/A Biolegend 302943 5μg/ml
Anti-human CD45 Confocal HI30 Unconjugated Proteintech CL647-65109 1 in 200
Anti-human HSP60 Confocal 4B9/89 Unconjugated ThermoFisher MA3-012 1 in 50

Murine CD4+ T cell Analysis
Anti-mouse CD3 Cell Culture 145-2C11 N/A Biolegend 100340 1μg/ml
Anti-mouse CD28 Cell Culture 37.51 N/A Biolegend 102121 5μg/ml
Anti-mouse IFN-γ FACS XMG1.2 FITC Biolegend 505806 1 in 100
Anti-mouse TNF-α FACS MP6-XT22 PE Biolegend 506306 1 in 200
Anti-mouse IL-2 FACS JES6-5H4 BV421 Biolegend 503826 1 in 100
Anti-mouse CD4 FACS GK1.5 APC Biolegend 100412 1 in 200
Anti-mouse CD4 FACS GK1.5 AF-700 Biolegend 100429 1 in 200
Anti-mouse CD8 FACS 53-6.7 PE-CY7 Biolegend 100722 1 in 200
Anti-mouse CD25 FACS PC61 AF-700 Biolegend 102024 1 in 200
Anti-mouse CD45 FACS 30-F11 BV605 Biolegend 103139 1 in 200
Anti-mouse CD45.1 FACS A20 Percp5.5 Biolegend 110728 1 in 200
Anti-mouse CD45.2 FACS 104 BV421 Biolegend 109832 1 in 200
Anti-mouse CD69 FACS H1.2F3 BV605 Biolegend 104529 1 in 200
Anti-mouse PD-1 FACS 29F.1A12 PE Biolegend 135205 1 in 200
Anti-mouse TCRβ FACS H57-597 PerCP/Cy5.5 Biolegend 109228 1 in 200
Anti-mouse CD19 FACS 6D5 PE Biolegend 115507 1 in 200
Anti-mouse FOXP3 FACS MF-14 BV421 Biolegend 126419 1 in 50
Anti-mouse CD62L FACS MEL-14 APC/CY7 Biolegend 104427 1 in 200
Anti-mouse IL-17 FACS TC11-18H10.1 PE-Cy7 Biolegend 506921 1 in 200
Anti-mouse RORγt FACS B2D PE ebioscience 12698180 1 in 200
Anti-puromycin FACS 2A4 AF488 Biolegend 381606 1 in 100
Anti-γH2AX FACS 2F3 FITC Biolegend 613404 1 in 100
Poly (ADP-ribose) (PAR) detection reagent WB N/A N/A Merck MABE1031 1 in 1000
Reagents used for both human and murine CD4+ T cell analysis
Fixable viability dye eFluor™ 780 FACS N/A APC-Cy7 Invitrogen 65-0865-14 1 in 1000
Zombie violet fixable viability kit FACS N/A BV421 Biolegend 423114 1 in 500
NFAT1 XP(R) Rabbit mAb FACS D43B1 AF647 Cell Signalling Technology 14201S 1 in 50
Donkey anti-rabbit IgG (H+L) FACS Polyclonal AF555 Invitrogen A31572 1 in 500
Donkey anti-rabbit IgG (min. x-reactivity) FACS Polyclonal BV421 Biolegend 406410 1 in 100

Validation

Antibodies used for flow cytometry were well established and validated by suppliers. They were additionally validated in our study by use of fluoresence minus one (FMO) controls or secondary antibody only controls.
Antibodies used for Western Blot were validated by suppliers.

Animals and other research organisms

Policy information about studies involving animals; ARRIVE guidelines recommended for reporting animal research, and Sex and Gender in Research

Laboratory animals	Mouse, C57Bl/6, 6-8 weeks
Wild animals	The study did not use wild animals
Reporting on sex	Experiments with immune cells from mice, or in vivo experiments performed in mice, always involved both sexes within each litter. Our findings are therefore applicable to both sexes.
Field-collected samples	The study did not involve samples collected from the field.
Ethics oversight	All animal studies were approved by the United Kingdom Home Office (Project Licenses PP5876109 (SD) and PP1902420 (EWR).

Note that full information on the approval of the study protocol must also be provided in the manuscript.

Plants

Seed stocks

Report on the source of all seed stocks or other plant material used. If applicable, state the seed stock centre and catalogue number. If plant specimens were collected from the field, describe the collection location, date and sampling procedures.

Novel plant genotypes

Describe the methods by which all novel plant genotypes were produced. This includes those generated by transgenic approaches, gene editing, chemical/radiation-based mutagenesis and hybridization. For transgenic lines, describe the transformation method, the number of independent lines analyzed and the generation upon which experiments were performed. For gene-edited lines, describe the editor used, the endogenous sequence targeted for editing, the targeting guide RNA sequence (if applicable) and how the editor was applied.

Authentication

Describe any authentication procedures for each seed stock used or novel genotype generated. Describe any experiments used to assess the effect of a mutation and, where applicable, how potential secondary effects (e.g. second site T-DNA insertions, mosiacism, off-target gene editing) were examined.

Flow Cytometry

Plots

Confirm that:

☐ The axis labels state the marker and fluorochrome used (e.g. CD4-FITC).

☒ The axis scales are clearly visible. Include numbers along axes only for bottom left plot of group (a 'group' is an analysis of identical markers).

☒ All plots are contour plots with outliers or pseudocolor plots.

☒ A numerical value for number of cells or percentage (with statistics) is provided.

Methodology

Sample preparation

Human CD4+ T cells were isolated from human peripheral blood by density-gradient centrifugation followed by positive selection using human CD4 Microbeads (Miltenyi, Cat# 130-045-101), purity was typically >95%. Cells were cultured at a density of 1 x 10^6 cells/ml in (unless otherwise indicated) RPMI-1640 containing 10% foetal calf serum (FCS) (Sigma Aldrich, Cat# F9665), 50 U/mL penicillin and 50 mg/mL streptomycin (Thermo Fisher Scientific, Cat# 15140122), and 50 IU/mL rIL-2 (PeproTech, Cat# 200-02) (RPMI/FCS). Cells were activated with 12 µl/ml ImmunoCult Human CD3/CD28 T Cell Activator (STEMCell, Cat# 10991) or, where indicated, plate bound anti-CD3 and/or anti-CD28 antibodies.

Murine splenocytes, thymocytes and lymph node mononuclear cells were isolated by manual disruption of tissue into a paste followed by washing twice with RPMI/FCS and filtration through a 70µM filter. Red blood cells were lysed in RB lysis buffer (Invitrogen, Cat# 00-4333-57)) on ice for 2 minutes before washing in PBS. Total CD4+ T cells were isolated from murine splenocytes by positive selection using mouse CD4 Microbeads (Miltenyi, Cat# 130-117-043), purity was typically >95%. Cells were cultured at a density of 1 x 106 cells/ml in (unless otherwise indicated) RPMI/FCS supplemented with 50µM β-mercaptoethanol (Gibco, Cat# 21985023). Cells were activated with plate bound anti-CD3 and anti-CD28 antibodies.

For analysis of cell surface protein expression, human and murine cells were stained in buffer containing 1x PBS and 2% FCS (FACS Buffer) with specific monoclonal antibodies for 30 minutes at 4°C then washed twice with FACS buffer prior to analysis. Cells were stained with Fixable Viability Dye (Table S1) during staining to assess cell viability.

For assessment of intracellular protein expression, cells were stained for viability and cell surface protein expression as before, washed twice with FACS buffer, then fixed using FoxP3 fixation/permeabilization solution (eBioscience, Cat# 005523-00) for 20 minutes at 4°C. Cells were washed once with FoxP3 permeabilization buffer and incubated with antibodies for 30-60 minutes. After washing a further two times cells were either analysed or incubated with secondary donkey anti-Rabbit antibody IgG1 for 20minutes at room temperature. Cells were then again washed twice before analysis.

For analysis of intracellular cytokine abundance, cells were first activated for 4 hours with Cell Activation Cocktail with Brefeldin A (BioLegend Cat# 423,304) prior to fixation, permeabilization and intracellular staining as described above.

Instrument

BD LSR Fortessa and Beckman Coulter CytoFLEX S

Software

BD FACSDiva, Beckman Coulter CytExpert, FlowJo V10.

Cell population abundance

We did not undertake flow cytometry cell sorting. For flow cytometry analysis, population frequencies are detailed within the example gating strategies provided.

Gating strategy

All gating strategies are provided in the Supplementary Material.

☒ Tick this box to confirm that a figure exemplifying the gating strategy is provided in the Supplementary Information.
