# Supplementary figures and images for: Cytoplasmic NAD/H synthesis via NRK1 regulates inflammatory capacity and promotes survival of CD4+ T cells
Source: Nat Commun. 2026 Feb 4;17:2349. doi: 10.1038/s41467-026-68863-w (PMC12979809; doi:10.1038/s41467-026-68863-w)

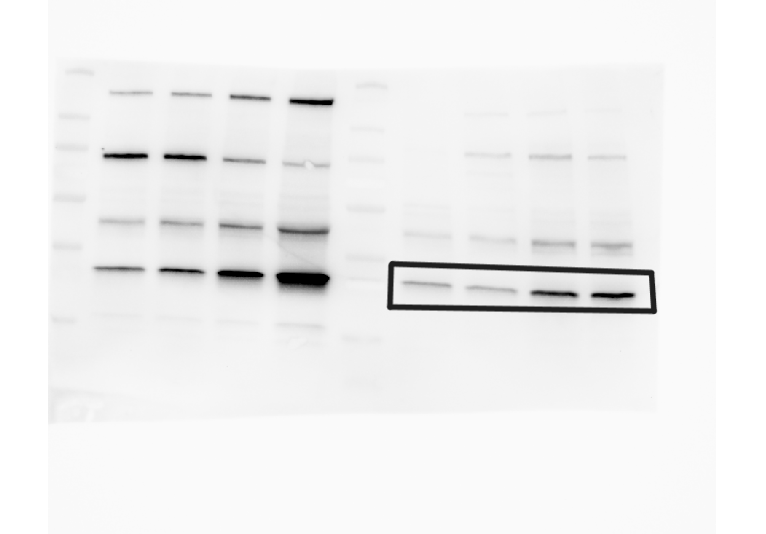

Supplement: Supplementary file 5 — Source Data 2 [file 41467_2026_68863_MOESM5_ESM.tif]

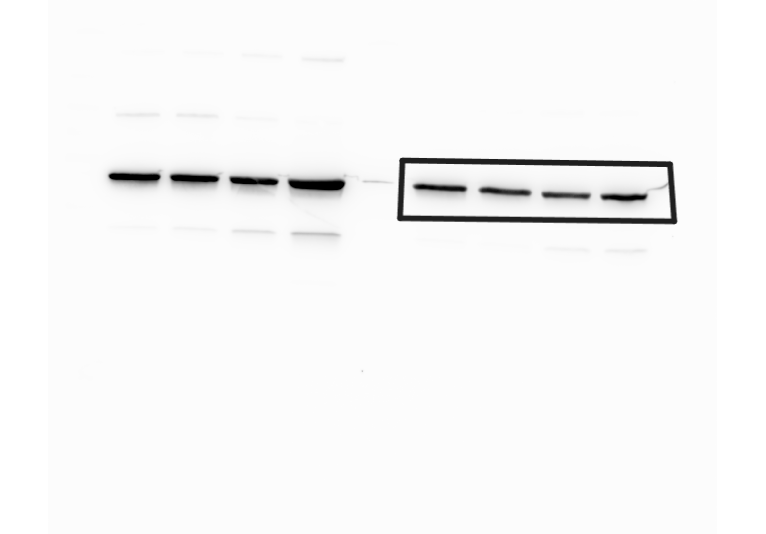

Supplement: Supplementary file 6 — Source Data 3 [file 41467_2026_68863_MOESM6_ESM.tif]
